# A Case of Priapism

**Published:** 1883-12

**Authors:** A. H. Boys


### A CASE OF PRIAPISM. By A. H. Boys, L.R.C.P.

W. S., æt. 65, gardener, came to see me on the 12th May, 1883, complaining of an erection of the penis which had already lasted a week.

The history was that while riding a horse it ran away with him, and the unusual exertion had rubbed the skin off the nates, there was also considerable bruising of the gluteal region generally. The next morning he had an erection (a rarity with him) which had lasted ever since. He had had sexual intercourse in the hopes it would disappear, but that seemed to make it worse; he also suffered a good deal of pain. On examination I found the whole penis swollen, red and œdematous, and it looked at first sight as if he had a paraphimosis; this was in consequence of the foreskin (which was naturally retracted) at the base of the glans being so puffy and œdematous.

I at first ordered a cold lead lotion to be constantly applied, and gave him a mixture containing ten grains of bromide of potassium every three hours. In five days there was no improvement. I then pricked the penis all over with a fine needle; serum flowed abundantly, so I ordered hot bathing, poultices, &c., hoping to encourage the flow of serum, and so reduce the tension on the whole organ.

There was nothing to account for this erection apparently beyond the slight injury to the nates before mentioned. His bowels were thoroughly cleared out by aperients of calomel and jalap; there was no swelling to be felt per rectum, or by palpation anywhere in the pelvis.

The man is temperate; rather prematurely aged. He had a slight attack of bronchitis, also slight hemiplegia about six months before, but there are no signs of paralysis now, and with the exception of the persistent erection of the penis, together with the pain in that organ, and consequent irritability of temper, he is quite well.

The hot treatment failing to produce relief, I again applied cold, in the shape of ice, to the penis and scrotum, a mustard plaister to the spine, giving him internally ten minims of tincture of belladonna every three hours. On the 22nd May, ten days after he came to me, Dr. Shingleton Smith saw him with me. He suggested belladonna suppositories, each containing half a grain of that drug. One was ordered to be inserted every four hours. I visited him on the 24th, when he assured me he could not use the suppositories as they caused so much pain, but on more minute enquiry I discovered to my horror he had been vainly struggling to push a large suppository down his penis!

From this date, however, they were used properly and persistently, in conjunction with a pill containing two grains of iodoform and a grain and a half of extract of conium. In about three days there was a decided improvement, the penis gradually recovered its normal condition, till at the end of three weeks there was no trace of its recent turbulence.

---

